# Post-insufflation diaphragm contractions in patients receiving various modes of mechanical ventilation

**DOI:** 10.1186/s13054-024-05091-y

**Published:** 2024-09-18

**Authors:** Antenor Rodrigues, Fernando Vieira, Michael C. Sklar, L. Felipe Damiani, Thomas Piraino, Irene Telias, Ewan C. Goligher, W. Darlene Reid, Laurent Brochard

**Affiliations:** 1 Keenan Centre for Biomedical Research, Li Ka Shing Knowledge Institute, Unity Health Toronto, Toronto, ON Canada; 2 Interdepartmental Division of Critical Care Medicine, University of Toronto, Toronto, ON Canada; 3 Escuela de Ciencias de La Salud, Facultad de Medicina, Pontificia Universidad Catolica de Chile, Santiago, Chile; 4 Division of Respirology, Department of Medicine, University Health Network and Mount Sinai Hospital, Toronto, ON Canada; 5 Department of Physiology, University of Toronto, Toronto, Canada; 6 Department of Physical Therapy, University of Toronto, Toronto, Canada; 7 KITE, Toronto Rehabilitation Institute, University Health Network, Toronto, Canada; 8 Department of Anesthesia, McMaster University, Hamilton, ON Canada; 9 St. Michael’s Hospital, Room 4-709, 36 Queens St E, Toronto, M5B 1W8 Canada

**Keywords:** Respiration, Artificial, Patient-ventilator asynchrony, Diaphragm, Muscle contraction

## Abstract

**Background:**

During mechanical ventilation, post-insufflation diaphragm contractions (PIDCs) are non-physiologic and could be injurious. PIDCs could be frequent during reverse-triggering, where diaphragm contractions follow the ventilator rhythm. Whether PIDCs happens with different modes of assisted ventilation is unknown. In mechanically ventilated patients with hypoxemic respiratory failure, we aimed to examine whether PIDCs are associated with ventilator settings, patients’ characteristics or both.

**Methods:**

One-hour recordings of diaphragm electromyography (EAdi), airway pressure and flow were collected once per day for up to five days from intubation until full recovery of diaphragm activity or death. Each breath was classified as mandatory (without-reverse-triggering), reverse-triggering, or patient triggered. Reverse triggering was further subclassified according to EAdi timing relative to ventilator cycle or reverse triggering leading to breath-stacking. EAdi timing (onset, offset), peak and neural inspiratory time (Ti_neuro_) were measured breath-by-breath and compared to the ventilator expiratory time. A multivariable logistic regression model was used to investigate factors independently associated with PIDCs, including EAdi timing, amplitude, Ti_neuro_, ventilator settings and APACHE II.

**Results:**

Forty-seven patients (median[25%-75%IQR] age: 63[52–77] years, BMI: 24.9[22.9–33.7] kg/m^2^, 49% male, APACHE II: 21[19–28]) contributed 2 ± 1 recordings each, totaling 183,962 breaths. PIDCs occurred in 74% of reverse-triggering, 27% of pressure support breaths, 21% of assist-control breaths, 5% of Neurally Adjusted Ventilatory Assist (NAVA) breaths. PIDCs were associated with higher EAdi peak (odds ratio [OR][95%CI] 1.01[1.01;1.01], longer Ti_neuro_ (OR 37.59[34.50;40.98]), shorter ventilator inspiratory time (OR 0.27[0.24;0.30]), high peak inspiratory flow (OR 0.22[0.20;0.26]), and small tidal volumes (OR 0.31[0.25;0.37]) (all *P* ≤ 0.008). NAVA was associated with absence of PIDCs (OR 0.03[0.02;0.03]; *P* < 0.001). Reverse triggering was characterized by lower EAdi peak than breaths triggered under pressure support and associated with small tidal volume and shorter set inspiratory time than breaths triggered under assist-control (all *P* < 0.05). Reverse triggering leading to breath stacking was characterized by higher peak EAdi and longer Ti_neuro_ and associated with small tidal volumes compared to all other reverse-triggering phenotypes (all *P* < 0.05).

**Conclusions:**

In critically ill mechanically ventilated patients, PIDCs and reverse triggering phenotypes were associated with potentially modifiable factors, including ventilator settings. Proportional modes like NAVA represent a solution abolishing PIDCs.

## Background

**Fig. 1:**
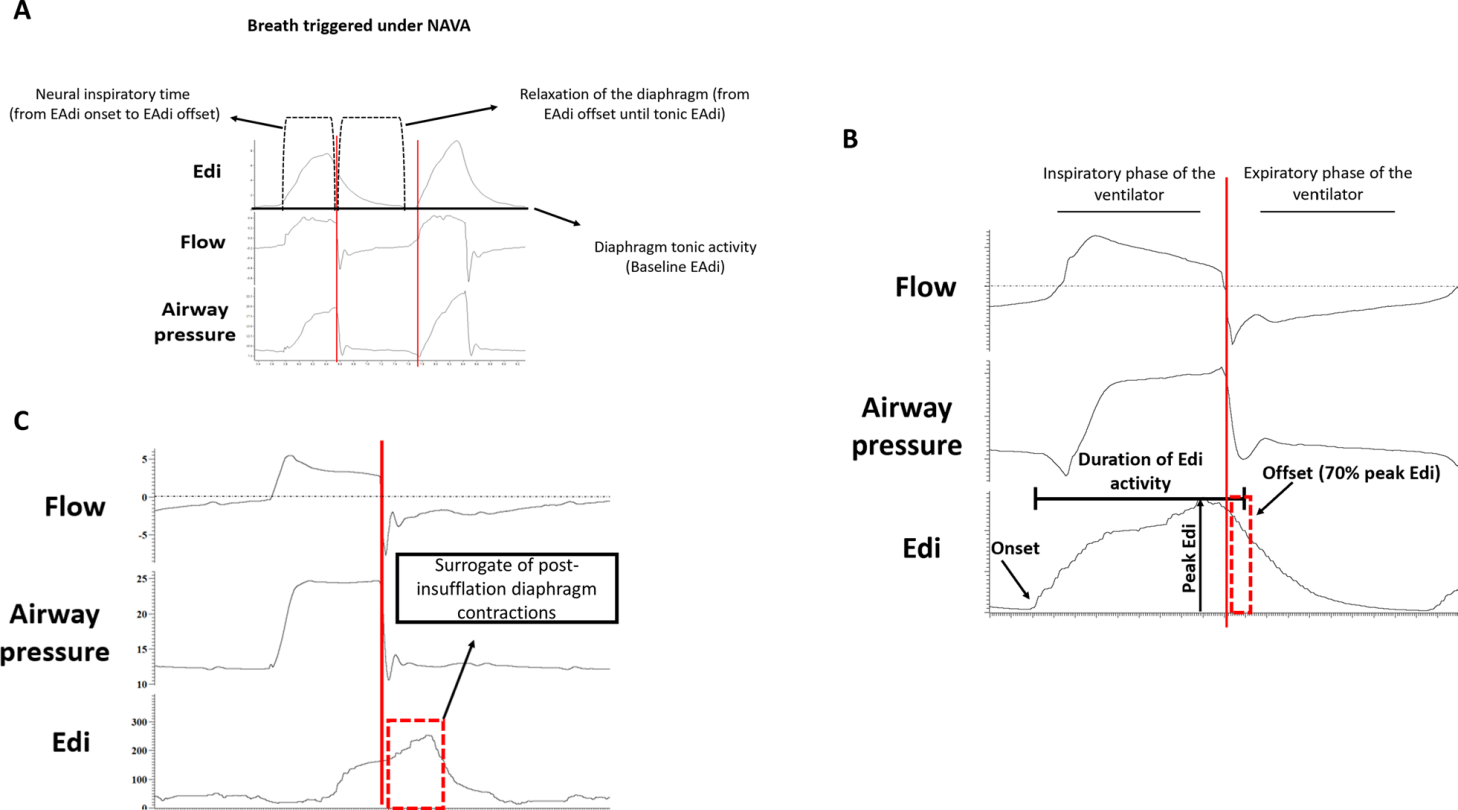
**A** Synchronous breaths triggered under NAVA with schematic representation of inspiratory neural time, active relaxation of the diaphragm during expiration and diaphragm tonic activity. **B** EAdi parameters example during a breath triggered under pressure controlled. Note that EAdi offset occurs during the expiratory time of the ventilator characterizing a post-insufflation diaphragm contraction. **C** example of a reverse triggering breath during pressure controlled with EAdi peak occurring during the expiratory phase of the ventilator as a surrogate of post-insufflation diaphragm contraction. Red dashed lines highlight post-insufflation diaphragm contraction

During each breath, diaphragm activity progressively increases, reaching its maximum contraction during inspiration before relaxing during early expiration and returning to its tonic level by the end of expiration (Fig. 1A) [1]. However, in mechanically ventilated patients, a non-physiologic phenomenon can occur in the form of a mismatch between the insufflation time of the ventilator and the neural inspiratory time of the patient, leading to patient-ventilator dyssynchrony. Consequently, the maximum contraction of the diaphragm and the end of neural inspiratory time can occur post-insufflation, during the expiratory phase of the ventilator. This phenomenon is henceforth referred to as *post-insufflation diaphragm contractions* (PIDCs). This is particularly evident during reverse triggering (Table 1 and Fig. 1) [2–4].

**Table 1:** Definitions

Type of breaths	Criteria
PIDCs	When the maximum contraction of the diaphragm and/or the end of neural inspiratory time occur post-insufflation, during the expiratory phase of the ventilator. It can occur during controlled ventilation due to reverse triggering dyssynchrony or during assisted ventilation, when the patient triggers the mechanical insufflation
Breath	Mechanical ventilator cycle from the start of one mechanical insufflation until the next
Ventilator inspiratory time	Inspiratory time on the ventilator. In pressure support will be determined by the cycling off criteria and in assist-control or controlled ventilation will be set on the ventilator (pressure controlled) or determined by the flow and volume set (volume control)
Mandatory breath without reverse triggering	Assist-control ventilation; Expiratory time of the previous breath equal to set expiratory time on the ventilator; No drop in the pressure signal or increase in the EAdi signal at the onset of the mechanical insufflation
Reverse triggering	Assist-control ventilation; Expiratory time of the previous breath equal to set expiratory time on the ventilator; EAdi not increasing above 0.5 µv at the onset of the mechanical insufflation; No drop in the airway pressure < -0.33 cmH_2_O at the onset of the mechanical insufflation; Increasing EAdi activity above 0.5 µv starting after the onset of the mechanical insufflation
Patient triggered breath	EAdi increasing above 0.5 µv or a drop in the airway pressure < − 0.33 cmH_2_O at the onset of the mechanical insufflation; Expiratory time of the previous breath can be shorter or equal to set expiratory time
Double cycling	A mechanical insufflation triggered by the patient due to a reverse triggering leading to breath stacking
Double triggering	Two consecutive mechanical insufflations triggered by the patient due to short cycling or high-inspiratory drive
*Reverse triggering phenotypes*	
Early reverse triggering with early relaxation	*EAdi onset* occurs during the ventilator inspiratory time *EAdi peak* and *EAdi offset* occurring during the ventilator inspiratory time
Early reverse triggering with delayed relaxation	*EAdi onset* occurs during the ventilator inspiratory time *EAdi peak* occurring during the ventilator inspiratory time *EAdi offset* occurring during the expiratory time of the ventilator
Mid-cycle reverse triggering	*EAdi onset* occurs during the ventilator inspiratory time *EAdi peak* occurs during the expiratory time of the ventilator
Late reverse triggering	*EAdi onset* occurs during the expiratory time of the ventilator
Reverse triggering leading to double cycling	*EAdi onset* occurs during the inspiratory time of the ventilator and triggers a second consecutive breath leading to reverse triggering with breath stacking

**Fig. 2:**
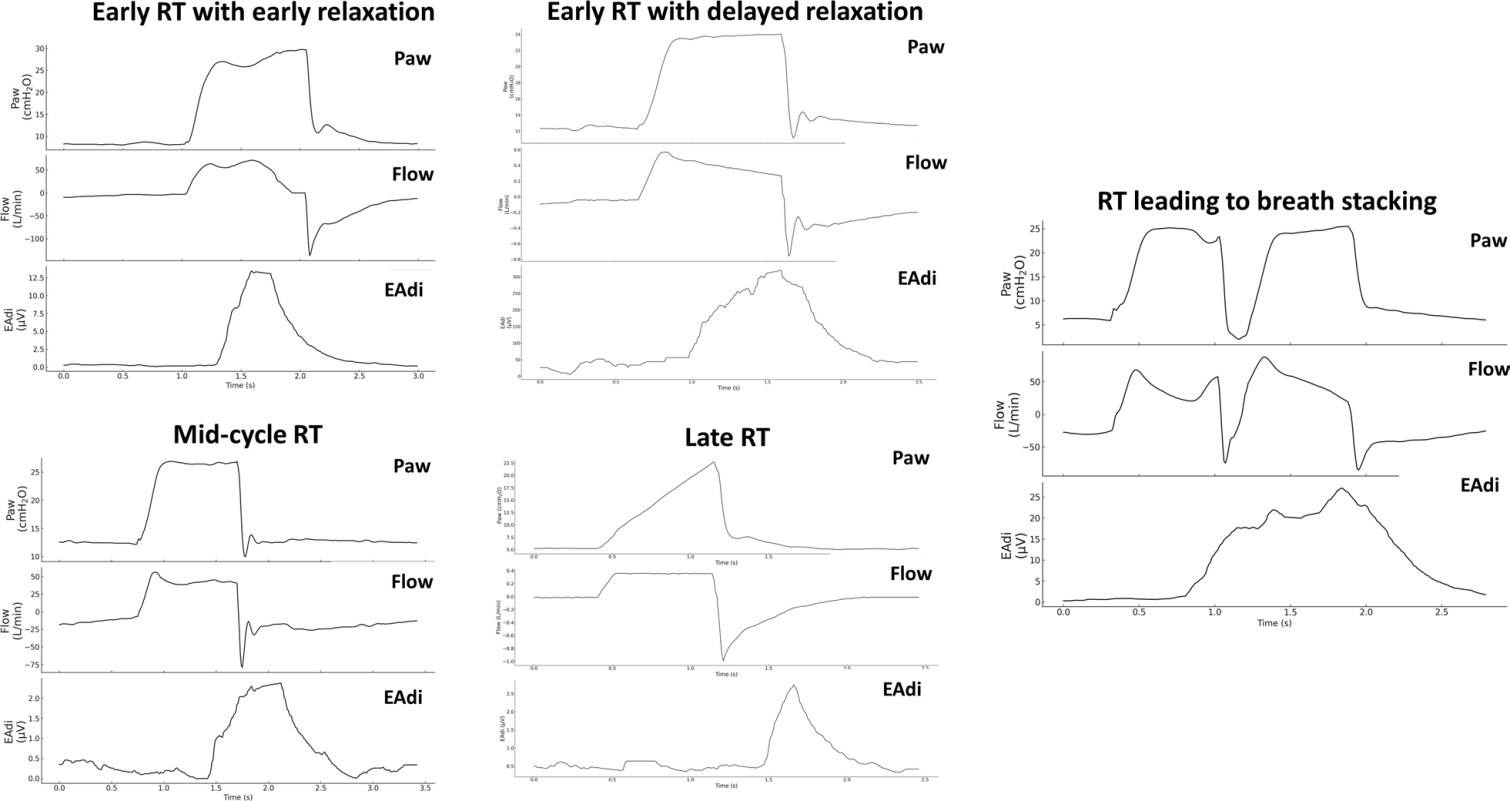
Reverse triggering phenotypes. RT: reverse triggering; Paw: airway pressure; EAdi: electrical activity of the diaphragm. Early reverse triggering with early relaxation: EAdi onset occurs during the ventilator inspiratory time and EAdi peak and EAdi offset occurring during the ventilator inspiratory time. Early reverse triggering with delayed relaxation: EAdi onset occurs during the ventilator inspiratory time, EAdi peak occurring during the ventilator inspiratory time and EAdi offset occurring during the expiratory time of the ventilator. Mid-cycle reverse triggering: EAdi onset occurs during the ventilator inspiratory time and peak EAdi occurs during the expiratory time of the ventilator. Late reverse triggering: EAdi onset occurs during the expiratory time of the ventilator. Reverse triggering leading to breath stacking: EAdi onset occurs during the inspiratory time of the ventilator and triggers a second consecutive breath leading to reverse triggering with breath stacking

Reverse triggering is a type of dyssynchrony that was first described in 2013[5]; however, it may have remained undetected since the dawn of mechanical ventilation[6]. Reverse triggering is characterized by a diaphragm contraction starting after the onset of a mandatory mechanical insufflation[3, 5]. Different *reverse triggering phenotypes* have been described based on the timing of the diaphragm contraction in relation to the phase of the ventilator cycle (Table 1 and Fig. 2)[3, 7]. For instance, during *early reverse triggering with early relaxation* the diaphragm contraction starts soon after the insufflation and its maximum contraction occurs during the inspiratory phase of the ventilator, while during *mid-cycle reverse triggering* the maximum diaphragm contraction occurs post-insufflation (i.e., PIDCs)[3, 7]. One animal model study where PIDCs occurred during reverse triggering suggested that, when coupled with small efforts, reverse triggering could contribute to the preservation of diaphragm function[8].

In assisted ventilation, where the patient triggers the ventilator, there is very little data describing whether PIDCs occur and their clinical consequences [4]. However recently, a prospective physiological cohort study in ICU patients under mechanical ventilation suggested that PIDCs may contribute to ventilator-induced diaphragm dysfunction as measured by a reduction in neuromuscular coupling [4].

Physiological studies have shown that PIDCs occur when the thoracic volume is decreasing, and the diaphragm is lengthening, which can lead to eccentric diaphragm contractions [9–13]. In an animal model inducing eccentric diaphragm contractions via bilateral supramaximal stimulation (large efforts), these efforts were associated with long lasting deterioration of diaphragm function that appeared to be partly due to structural damage [9]. Furthermore, PIDCs can also cause pendelluft (movement of air between lung regions), leading to overstretch of lung regions and lung injury [14, 15].

There is currently no direct evidence regarding the optimal strategy for managing PIDCs, independently if PIDCs are occurring during reverse triggering or assisted ventilation. As for many dyssynchronies, clinicians often resort to increasing sedation depth or sometimes administering neuromuscular blocking agents [3, 16, 17]. However, paralysis or deep sedation are associated with diaphragm inactivity, diaphragm dysfunction, longer ICU stays, and potentially increased mortality [13, 18–21]. Understanding the factors associated with the occurrence of PIDCs, independently if PIDCs occurring during reverse triggering or assisted ventilation, will aid in developing safe strategies to mitigate potential harm and optimize associated benefits. Furthermore, because PIDCs are particularly evident during specific reverse triggering phenotypes [2–4], understanding factors associated with the different reverse triggering phenotypes could be hypothesis generating for future studies to aid developing strategies to manage the occurrence of reverse triggering to mitigate potential harm and optimize associated benefits.

Our hypothesis was that PIDCs would be mainly found associated with reverse triggering but could be found more rarely with other assisted modes; and second that factors related to the breath characteristics as well as factors related to ventilatory settings, and thus potentially modifiable, would be associated with reverse triggering. Our main objective was to determine the characteristics of patients’ respiratory drive and breathing pattern (e.g., neural inspiratory time, peak EAdi) as well as ventilator settings (e.g., set inspiratory time, rate and tidal volume) associated with the presence of PIDCs during both reverse triggering and assisted ventilation.

## Methods

### Study design

This is a secondary analysis of the DIVIP study [18], which was approved by the St. Michael’s Hospital Ethics Committee (REB#15–073) and registered on ClinicalTrials.gov (NCT02434016). The DIVIP study was conducted at the St. Michael’s Hospital from June 2015 to August 2017. It was a prospective observational study aimed at detecting the timing of resumption of clinically meaningful diaphragm activity after intubation in critically ill patients [18]. Herein, we used data from the DIVIP study to explore the characteristics of patients’ respiratory drive and breathing pattern as well as ventilator settings associated with the presence of PIDCs during both reverse triggering and assisted ventilation.

#### Patients

Adult patients intubated in the intensive care unit (ICU) or emergency department, expected to have a duration of mechanical ventilation > 48 h and an oro- or nasogastric feeding tube equipped with electrodes at the level of the diaphragm (EAdi catheter, Getinge, Solna, Sweden) placed within 30 min after intubation. Daily sedation interruption or minimization and spontaneous breathing trials were performed as per the University of Toronto academic ICU policy [22]. Patients were enrolled between June 2015 and August 2017.

### Data collection

The EAdi catheter was positioned as per standard practice, using the distance from the nose to the ear lobe to the xiphoid process of the sternum [23], and connected to a Servo-I ventilator (Getinge, Solna, Sweden) equipped with a Neurally Adjusted Ventilator Assist (NAVA) module for EAdi recordings. The catheter positioning was carefully adjusted by visualizing a decrease in the QRS complex amplitude from top to bottom traces and the P wave disappearing on the bottom tracing depicted on the ventilator’s screen. This approach ensures a standardized positioning and homogeneous EAdi amplitude as previously reported [23].

1-h waveform recordings of EAdi, airway pressure and flow were obtained once per day from the ventilator at a fixed time of the day each day, from intubation until full recovery of diaphragm activity, extubation, death or 5 days. Thus, each patient could have from one up to a maximum of five recording days. For the statistical analysis (see below), each recording was then assigned a unique ID number based on the patient ID and study day (e.g., recording 1.1 represented a recording from patient ID 1, performed on study day 1). Waveform recordings (100 Hz) of EAdi, airway pressure and flow were obtained by connecting the ventilator to a laptop via a RS232 cable using a dedicated signal acquisition software (Servo Tracker, Getinge, Solna, Sweden). Data were stored for off-line analysis in a software routine developed in R.

Baseline patient characteristics included age, sex, body mass index (BMI), Acute Physiology and Chronic Health Evaluation Score II (APACHE) and reason(s) for intubation were collected from the hospital electronic records.

### Offline analysis

#### Breath type classification

Each breath was classified by off-line automated signal analysis according to pre-specified definitions as previously described and listed in Table 1 [2, 24]. For quality control, randomly selected breaths were visually inspected to confirm the accuracy of the off-line automated signal analysis. Each breath was classified as: (i) mandatory breath without reverse triggering, (ii) reverse triggering breath, (iii) patient triggered breath, (iv) breath stacking occurring because of reverse triggering, or (v) breathing stacking because of the same patient effort) based on EAdi, airway pressure and flow waveforms according to criteria described before [2, 3, 24]. Details are shown in Table 1. Reverse triggering was further subclassified according to EAdi timing relative to ventilator cycle. Phenotypes of reverse triggering have been previously described based mainly on the timing of onset (Table 1) [3]. We used the classification presented in Table 1 and separating reverse triggering breaths as follow:

(i) early reverse triggering with early relaxation.
(ii) early reverse triggering with delayed relaxation.
(iii) mid-cycle reverse triggering.
(iv) late reverse triggering.
(v) reverse triggering leading to breath stacking.

### Breath

#### EAdi parameters

The onset of the EAdi activity (i.e., onset of patient inspiratory effort) was defined by a significant EAdi increase of at least 0.5 µv above the baseline [25]. EAdi offset was defined as when the EAdi signal dropped to 70% of its peak [25, 26]. The diaphragm activity and its duration, i.e., neural inspiratory time (Fig. 1A) was defined as the time (in seconds) elapsed from the onset to the offset of the EAdi activity [27]. The rest of EAdi (after the offset) was considered as relaxation. Peak EAdi (in µv) was defined as the highest EAdi value during each patient’s inspiratory effort (i.e., between EAdi onset and EAdi offset) (Fig. 1B). We calculated the EAdi slope as the EAdi peak divided by the duration of the EAdi activity.

#### Identification of breaths with PIDCs contraction

PIDCs were defined as when the EAdi offset occurred during the expiratory time of the ventilator, i.e.,70% of the peak (Fig. 1C).

#### Ventilator parameters

From the ventilator waveforms we calculated breath-by-breath the ventilator inspiratory (insufflation plus pause, if set) and expiratory time (in seconds), peak and mean inspiratory flow (L/s), expired tidal volume (in ml and ml/kg of predicted body weight), respiratory rate (breaths per minute [bpm]) and positive end-expiratory pressure (PEEP). Inspired fraction of oxygen (FIO_2_) was recorded from the hospital electronic system. We also analyzed patient triggered breaths in assist-control (volume or pressure), pressure support ventilation and neurally adjusted ventilatory assist (NAVA).

#### Reverse triggering characteristics

The strength of the phase-locking of reverse triggering breaths was measured with the phase angle (θ) [3]:

$${\uptheta } \left(^\circ \right) = \frac{Inspiratory\;effort\;onset\;time - mechanical\;insufflation\;onset\;time}{{mechanical\;ventilator\;cycle\;duration}}*360^\circ$$

where the inspiratory effort onset time is the EAdi onset time and the mechanical ventilator cycle duration is the time elapsed between the onset of two mechanical insufflations.

Respiratory entrainment was defined as the ratio of reverse triggering for each mechanical insufflation [3]: 1:1 entrainment pattern when one reverse triggering breath occurred with one mechanical insufflation over three or more consecutive mechanical insufflations, and 1:2 entrainment pattern when one reverse triggering occurred with every other mechanical insufflation for at least six consecutives mechanical insufflations.

### Statistical analysis

**Fig. 3:**
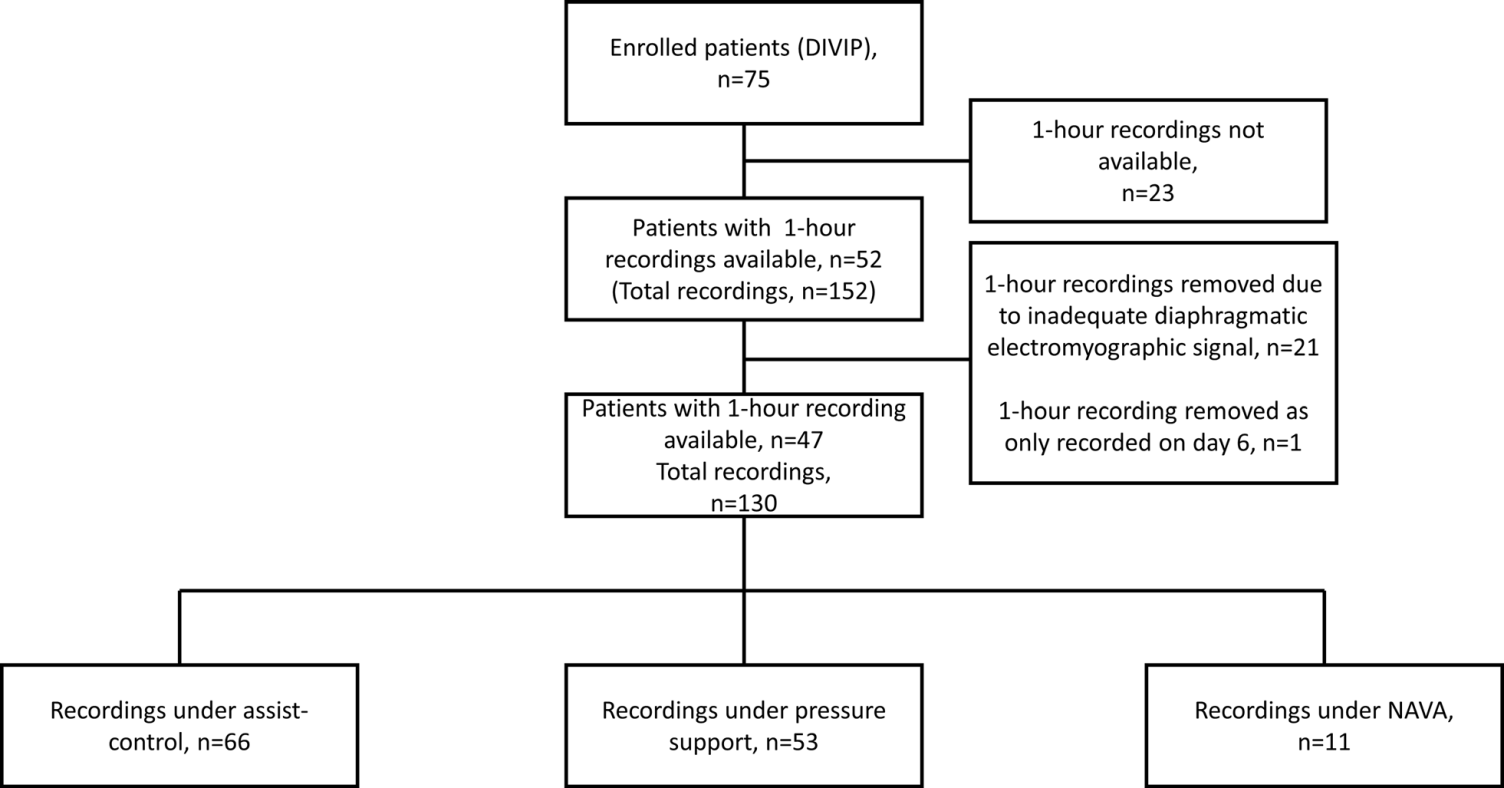
Flow chart

As this is a secondary analysis of a prospective observational study, we included all patients with available data and meeting our inclusion criteria (Fig. 3). Continuous data were reported as median [IQR 25–75%] and categorical data as frequencies and percentages. Our analyses considered breaths as the unit of analysis.

We compared EAdi and ventilatory parameters between reverse triggering, breaths triggered under assist-control and breaths triggered under pressure support with mixed-models using the types of breath as fixed factor and patient ID as random factor. To compare EAdi and ventilatory parameters between breaths with vs. without PIDCs, we additionally included the presence/absence of PIDCs as a fixed factor in the mixed model.

**Fig. 4:**
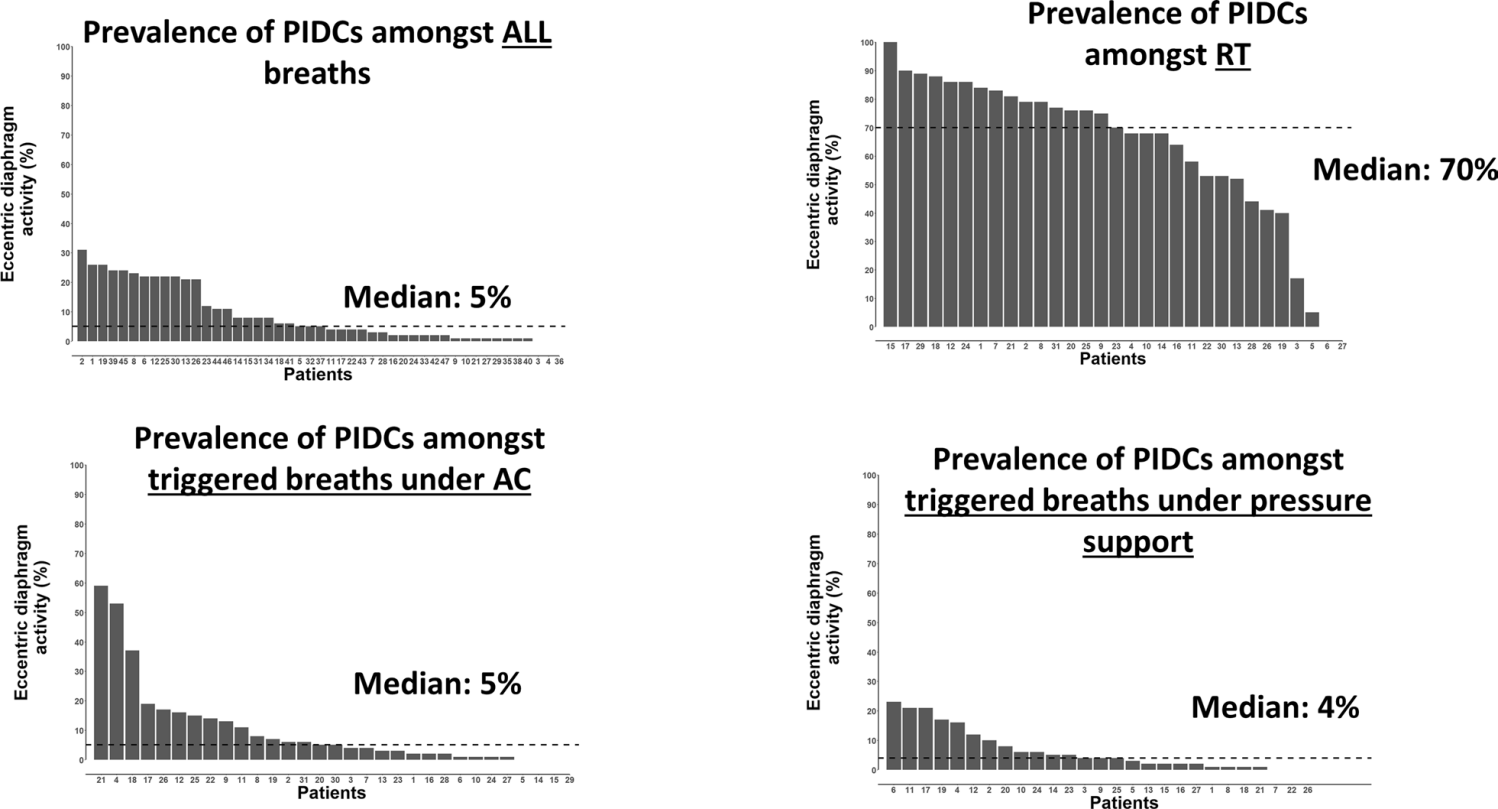
Prevalence of post-insufflation diaphragm contraction (PIDCs) in each patient according to the mode of ventilation. Five patients were ventilated with NAVA

We built histograms to show the prevalence of breaths with PIDCs in each patient in all recordings (Fig. 4A) and according to each mode of ventilation (Fig. 4B–E).

We further compared EAdi and ventilatory parameters and reverse triggering characteristics between the different phenotypes of reverse triggering with mixed models using reverse triggering phenotype as a fixed factor and patient ID as a random factor.

We investigated if EAdi parameters and ventilator settings were independently associated with PIDCs with multivariable logistic model. The presence of PIDCs was used a dichotomous variable and parameters included both patient (e.g., peak EAdi and neural inspiratory time, APACHE II, age) and ventilator characteristics (e.g., ventilator mode, ventilator inspiratory time, peak inspiratory flow, tidal volume).

*P* < 0.05 was considered as the statistically significant threshold for all analysis.

## Results

### Patient characteristics

Figure 3 shows the study flow chart. Forty-seven out of the 75 patients included in the DIVIP study had at least a 1-h recordings of EAdi, airway pressure and flow with good quality available and were included in this analysis. Patients age was (median [25–75% IQR]) 63 [52 -77], BMI kg/m^2^ 24.9 [22.9–33.7], 49% male, APACHE II 21 [19–28]. The reason for intubation was mostly pulmonary (24 patients). Other reasons included neurologic (9 patients), cardiac (3 patients) and other (11 patients).

### Prevalence of each type of breath

**Fig. 5:**
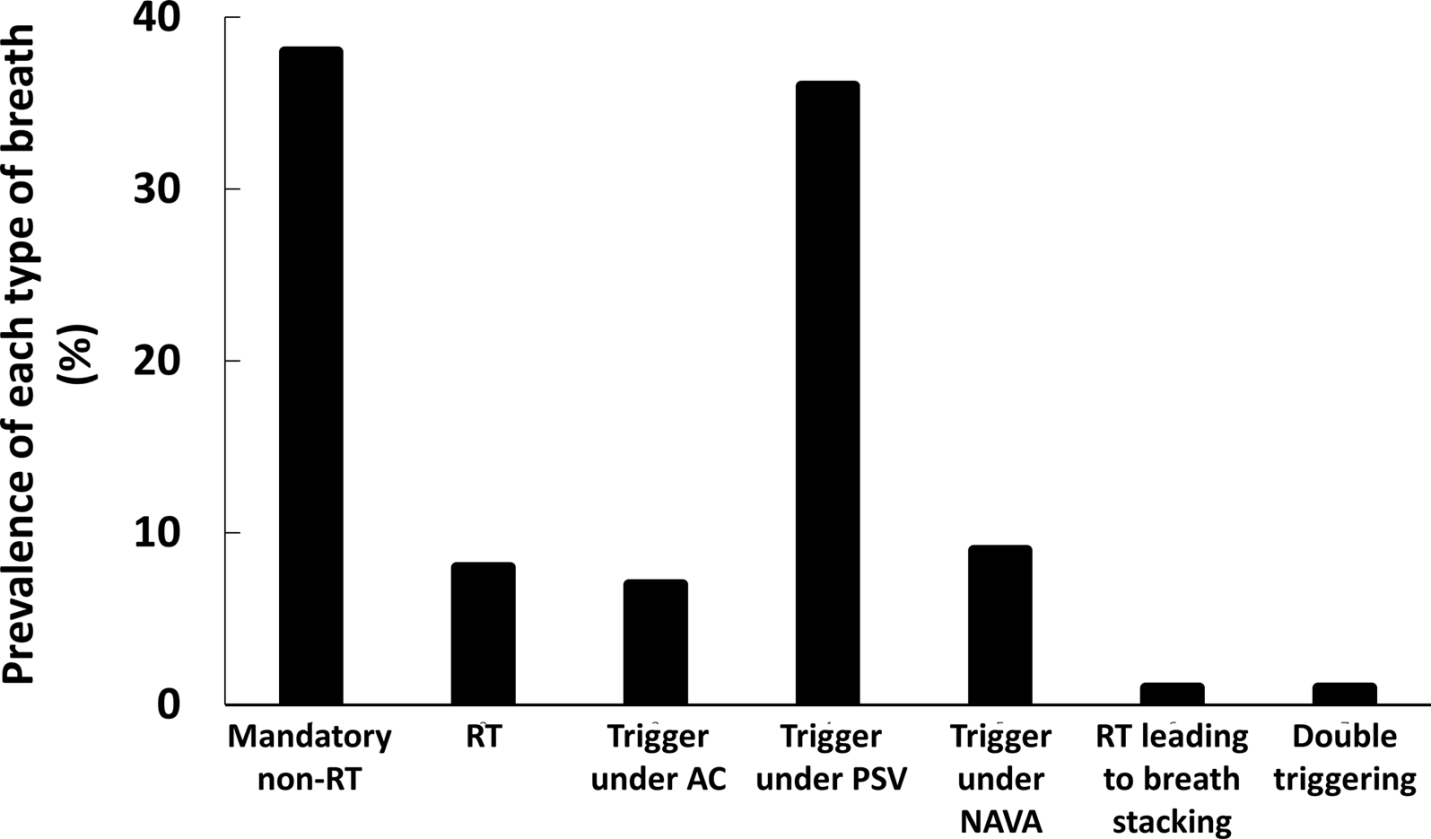
Prevalence of each type of breath during available recordings from the DIVIP study. RT: reverse triggering; AC: assist-control; PSV: pressure support ventilation; NAVA: neurally adjusted ventilatory assist. Mandatory non-RT: 70,814 (38%) of the breaths. RT: 14,948 (8%) if the breaths. Trigger under assist-control: 12,400 (7%) of the breaths. Triggered under NAVA: 16,401 (9%) of the breaths. Reverse triggering leading to breath stacking: 1887 (1%) of the breaths. Double triggering: 524 (< 1%) of the breaths

Each patient contributed with 2 ± 1 (ranging from 1 to 5) one-hour recording. A total of 183,962 breaths were analyzed. Mandatory breaths with no reverse triggering and breaths triggered under pressure support were the most prevalent types of breath amongst all analyzed breaths (38% and 36% respectively; Fig. 5). Overall, 8% of breaths (13% of all breaths under assist-control) were reverse triggering (Fig. 5) and 31 out of the 47 patients (66%) had at least some reverse triggering breaths. Entrainment ratio 1:1 and 1:2 was present in 25 (80%) and 23 (74%) of the 31 patients having reverse triggering, respectively.

### Prevalence of PIDCs

As illustrated in Fig. 4, PIDCs were mostly prevalent during reverse triggering (74% of all reverse triggering breaths). However, PIDCs also occurred in 21% and 27% of all triggered breaths under assist-control and pressure support, respectively (Table 2). PIDCs occurred in only 5% of breaths under NAVA.

**Table 2:** Breath-by-breath analysis of EAdi parameters according to each type of breath and the presence of post-insufflation diaphragm contraction

PIDCs	RT			Triggered under assist-control			Triggered under pressure support		
	ALL	NO PIDCs	PIDCs	ALL	NO PIDCs	PIDCs	ALL	NO PIDCs	PIDCs
n (%)	14,948	3832 (26)	11,116 (74)	12,400	9832 (79)	2568 (21)	66,988	49,030 (73)	17,958 (27)
EAdi peak, µV	3 [2–5]	3 [2–6]	3 [2–5]^£^	3 [1–7]*	3 [1–5]	11 [5–24]^£^	7 [4–13]*¥	7 [4–13]	9 [5–17]^£^
EAdi slope, µV/sec	6 [4–10]	8 [6–15]	5 [4–9]	7 [5–13] *	6 [4–10]	12 [6–21]^£^	10 [6–18] *¥	10 [6–17]	9 [5–19]^£^
duration of EAdi activity, sec	0.5 [0.4–0.7]	0.4 [0.3–0.5]	0.6 [0.4–0.8]^£^	0.7 [0.5–1.0]*	0.7 [0.5–0.8]	1.0 [0.7–1.2]^£^	0.8 [0.6–0.9]*¥	0.7 [0.6–0.9]	0.9 [0.8–1.1]^£^
duration of EAdi activity, %Ti	72 [50–96]	48 [38–63]	79 [60–102]^£^	88[60–133]*	79[54–103]	141[99–169]^£^	104[89–120]*¥	100[86–113]	118[101–135]^£^
dP, sec	0.56 [0.35–0.81]	0.35 [0.24–0.60]	0.60 [0.45–0.83]^£^	NA	NA	NA	NA	NA	NA

*P < 0.05 vs. reverse triggering breaths; ¥ P < 0.05 vs triggered under assist-control; £ P < 0.05 vs no post-insufflation diaphragm contractions

### EAdi characteristics and ventilator parameters associated with PIDCs during reverse triggering

Table 2 shows the differences in EAdi and Table 3 ventilator parameters between reverse triggered breaths with vs without PIDCs.

**Table 3:** Breath-by-breath analysis of ventilatory parameters according to each type of breath and the presence of post-insufflation diaphragm contractions

PIDCs	RT			Triggered under assist-control			Triggered under pressure support		
	ALL	NO PIDCs	PIDCs	ALL	NO PIDCs	PIDCs	ALL	NO PIDCs	PIDCs
n (%)	14,948	3832 (26)	11,116 (74)	12,400	9832 (79)	2568 (21)	66,988	49,030 (73)	17,958 (27)
Ventilator Ti, sec	0.74[0.64–0.89]	0.84[0.70–0.95]	0.70[0.64–0.85]^£^	0.80[0.70–0.90]*	0.80[0.70–0.94]	0.74[0.5–0.85]^£^	0.75[0.61–0.89]*¥	0.75[0.60–0.88]	0.76[0.61–0.99]^£^
Mean insp. Flow, L/sec	0.46[0.41–0.49]	0.44[0.41–0.48]	0.48[0.41–0.50]^£^	0.49[0.42–0.58]*	0.49[0.41–0.57]	0.56[0.48–0.69]^£^	0.45[0.39–0.54]*¥	0.46[0.39–0.56]	0.44[0.40–0.50]^£^
Peak insp. Flow, L/sec	0.76[0.54–0.88]	0.81[0.69–0.84]	0.73[0.54–0.88]^£^	0.81[0.66–0.94]*	0.75[0.64–0.93]	0.86[0.78–1.02]^£^	0.69[0.58–0.79]*^£^	0.70[0.58–0.80]	0.67[0.57–0.75]^£^
Vt, ml	368[332–409]	377[368–446]	358[327- 399]	447[395–507]*	446[395–510]	452[393–497]	394[298–462] *¥	397[307–460]	382[276–475]
Vt, ml/kg PBW	7[5–7]	7[5–8]	7[5–7]	7[6–8]	7[6–8]	7[7–9]	7[6–8]	7[6–8]	6[6–8]
set respiratory rate, bpm	28[20–30]	30[20–30]	26[20–30]^£^	24[18–30]*	24[18–30]	24[18–33]^£^	NA	NA	NA

^*^ P < 0.05 vs. reverse triggering breaths; ¥P < 0.05 vs triggered under assist-control; £ P < 0.05 vs no post-insufflation diaphragm contractions

#### EAdi parameters

Reverse triggering breaths with PIDCs compared to reverse triggering breaths without PIDCs were characterized by later onset of EAdi activity (i.e., greater phase angle) and longer duration of EAdi activity (Table 2).

#### Ventilator parameters

Reverse triggering breaths with PIDCs compared to reverse triggering breaths without PIDCs were associated with short ventilator inspiratory time, smaller tidal volume and lower set respiratory rate (Table 3).

### EAdi characteristics and ventilator parameters associated with PIDCs for breaths triggered under assist-control and pressure support

Table 2 shows the differences in EAdi and Table 3 ventilator parameters between breaths triggered under assist-control and pressure support with vs without PIDCs.

#### EAdi parameters

Breaths triggered under assisted control and pressure support with PIDCs compared to without PIDCs were characterized by high EAdi peak and slope and longer duration of EAdi activity (Table 2).

#### Ventilator parameters

Breaths triggered under assisted control with PIDCs compared to without PIDCs were associated with shorter inspiratory time, high inspiratory flow and smaller tidal volume (Table 3).

Breaths triggered pressure support with PIDCs compared to without PIDCs were associated with low inspiratory flow and smaller tidal volume (Table 3).

### PIDCs during NAVA

Under NAVA, breaths with PIDCs compared to breaths without PIDCs were characterized by lower EAdi peak and longer duration of neural inspiratory time (Table 4) and associated with longer inspiratory time, lower inspiratory flow and reduced tidal volume (Table 4).

**Table 4:** Breath-by-breath analysis of EAdi and ventilatory parameters according to each type of breath and the presence of post-insufflation diaphragm contractions during NAVA

PIDCs	NAVA		
	ALL	NO PIDCs	PIDCs
N (%)	16,401	15,606 (95%)	795 (5%)
EAdi peak, µV	10.1 [6.83–21.6]	10.4 [6.91–22.4]	7.19 [5.52–10.0]*
duration of E Adi activity, sec	0.72 [0.62–0.82]	0.71 [0.61–0.81]	0.91 [0.74–1.06]*
duration of EAdi activity, %Ti	100[0.98–103]	100[97–103]	115 [106–129]*
Ventilator Ti, sec	0.74 [0.60–0.83]	0.74 [0.60–0.82]	0.79 [0.62–0.91]*
Mean insp. Flow, L/sec	0.41 [0.35–0.70]	0.41 [0.35–0.72]	0.39 [0.32–0.47]
Peak insp. Flow, L/sec	0.60 [0.49–1.03]	0.60 [0.49–1.07]	0.54[0.47–0.69]*
Vt, ml	0.33 [0.26–0.57]	0.34 [0.26–0.58]	0.32 [0.28–0.39]*
set respiratory rate, bpm	NA	NA	NA

*P < 0.05 vs no post-insufflation diaphragm contractions

### Phenotypes of reverse triggering and EAdi and ventilator characteristics

The prevalence of each reverse triggering phenotype is described in Table 5. Reverse triggering leading to breath stacking represented 13% of all reverse triggering breaths; 80% of the reverse triggering leading to breath stacking were caused by mid-cycle reverse triggering while early reverse triggering caused the other 20%.

**Table 5:** Breath-by-breath analysis of EAdi parameters for different reverse triggering phenotypes

	Early RT w/early “relaxation”	Early RT w/late “relaxation”	Mid-cycle RT	Late RT	RT leading to DC
n (%)	3832 (26)	1207 (8)	4870 (33)	3558 (24)	1481 (10)
EAdi peak, µV	3 [2–6]	3 [2–4]*	3 [2–5]*	2 [2, 3]*Ŧ¥	7 [4–21]*Ŧ¥£
EAdi slope, µV/sec	8[6–15]	5[4–9]*	5[4–7]*	5[4–8]*	9[6–19]*¥£
duration of EAdi activity, sec	0.4[0.3–0.5]	0.5[0.4–0.7]*	0.6[0.6–0.8]*Ŧ	0.5[0.3–0.6]*¥	0.8[0.6–1.1]*Ŧ¥£
duration of EAdi activity, %Ti	48[38–63]	72[56–87]*	82[68–100] *Ŧ	65[45–90] *¥	108[84–160] *Ŧ¥£
dP, sec	0.35[0.24–0.60]	0.34[0.25–0.50]*	0.54[0.42–0.63] *Ŧ	1.0[0.8–1.37] *Ŧ¥	0.5[0.4–0.6] *Ŧ¥£

*P < 0.05 vs. early RT with early relaxation; Ŧ P < 0.05 vs. early RT with late relaxation; ¥ P < 0.05 vs. mid-cycle RT; £ P < 0.05 vs. late RT. PBW: predicted body weight

#### EAdi parameters

Early reverse triggering phenotype with early relaxation was characterized by higher EAdi slope, earlier onset and a shorter duration of EAdi activity than early reverse triggering with late relaxation, mid-cycle and late reverse triggering (Table 5). Reverse triggering leading to breath stacking was characterized with greater EAdi peak and EAdi slope and longer duration of EAdi activity than all other phenotypes (Table 5).

#### Ventilator parameters

Reverse triggering leading to breath stacking was associated with smaller tidal volumes than all other phenotypes (Table 6). Early reverse triggering was associated with the following ventilatory settings: longer ventilator’s inspiratory time, larger tidal volume and higher set respiratory rate than all other phenotypes (Table 6).

**Table 6:** Breath-by-breath analysis of ventilator parameters for different reverse triggering phenotypes

	Early RT w/early “relaxation”	Early RT w/late “relaxation”	Mid-cycle RT	Late RT	RT leading to DC
n (%)	3832 (26)	1207 (8)	4870 (33)	3558 (24)	1481 (10)
Ventilator Ti, sec	0.84[0.70–0.95]	0.70[0.50–0.90]*	0.74[0.60–0.90] *Ŧ	0.70[0.64–0.80] *Ŧ¥	0.70[0.65–0.89] *Ŧ¥
Mean Insp. Flow, L/sec	0.44[0.41–0.48]	0.48[0.42–0.64]*	0.47[0.39–0.49]*	0.48[0.42–0.50]*Ŧ¥	0.47[0.38–0.50]*¥
Peak Insp. Flow, L/sec	0.81[0.69–0.84]	0.75[0.56–0.93]*	0.74[0.54–0.88]*Ŧ	0.67[0.62–0.94] Ŧ¥	0.76[0.62–0.94]*¥£
Vt, ml	377[368–446]	369[356–416]	359[331–383]*Ŧ	354[324–417]*Ŧ¥	332[297–399]*Ŧ¥
Set respiratory rate, bpm	30[20–30]	28[20–30]*	24[20–30]*Ŧ	28[24–30]*Ŧ¥	28[20–34]*¥£

*P < 0.05 vs. early RT with early relaxation; Ŧ P < 0.05 vs. early RT with late relaxation; ¥ P < 0.05 vs. mid-cycle RT; £ P < 0.05 vs. late RT. PBW: predicted body weight

### Factors independent associated with PIDCs

Table 7 shows that both a higher amplitude and longer duration of the neural inspiratory drive on the one hand, and a shorter ventilator’s inspiratory time, a higher inspiratory flow and a lower tidal volume on the other hand were independently associated with the occurrence of PIDCs (all *P* ≤ 0.008). Longer neural inspiratory time had the strongest association with occurrence of PIDCs (OR [95%CI] 37.59[34.50–40.98), whereas be ventilated under NAVA had the strongest association with absence of PIDCs (OR[95%CI] 0.03 [0.02 to 0.03]; all *P* < 0.001; Table 7).

**Table 7:** Multivariable logistic model to investigate independent associations between EAdi parameters and ventilatory settings with the presence of post-insufflation diaphragm contractions (PIDCs)

	OR (95% CI)	ß (95% CI)	*P*
EAdi peak, µV	1.01 (1.01 to 1.01)	0.01 (0.01 to 0.01)	< 0.001
duration of EAdi activity, sec	37.59 (34.50 to 40.98)	3.63 (3.54 to 3.71)	< 0.001
Ventilator Ti, sec	0.27 (0.24 to 0.30)	− 1.31 (− 1.43 to − 1.19)	< 0.001
Peak insp. Flow, L/sec	0.22 (0.20 to 0.26)	− 1.49 (− 1.62 to − 1.36)	< 0.001
Vt, ml	0.31 (0.25 to 0.37)	− 1.18 (− 1.38 to − 0.99)	< 0.001
Ventilation mode (ref is assist-control)	NA		NA
Pressure support	0.16 (0.15 to 0.16)	− 1.85 (− 1.90 to − 1.81)	< 0.001
NAVA	0.03 (0.02 to 0.03)	− 3.60 (− 3.69 to − 3.52)	< 0.001
APACHE II	1.00 (1.00 to 1.01)	0.00 (0.00 to 0.01)	0.008
Age, years	0.98 (0.98 to 0.98)	− 0.02 (− 0.02 to − 0.02)	

Ti: inspiratory time; vt: tidal volume; NAVA: Neurally Adjusted Ventilatory Assist; APACHE II: acute physiology score of acute physiology and chronic health evaluation II; ß: Estimate; Std. Error: standard error; 95% CI: 95% confidence interval. Model created based on 62,694 breaths without PDCs plus 31,642 breaths with PIDCs

## Discussion

Our study shows that breaths with PIDCs are highly prevalent in mechanically ventilated patients with acute hypoxemic respiratory failure. They are much more common during reverse triggering, but also exist in about 20–25% of the breaths triggered by the patient during assist-control or pressure support ventilation. Proportional modes like NAVA represent a solution almost abolishing PIDCs. The amplitude and duration of the neural inspiratory time as well the ventilator inspiratory time, inspiratory flow and tidal volume were independently associated with PIDCs when analyzing all types of breaths in all modes together. Longer neural inspiratory time had the strongest association with PIDCs. During both reverse triggering and breaths triggered in assist-control ventilation, the presence of PIDCs was associated with low tidal volumes and shorter ventilator inspiratory time and characterized by a longer duration of neural inspiratory time. Additionally, PIDCs in breaths triggered under assist-control were associated with high inspiratory drive (high peak EAdi and EAdi slope). Mid-cycle reverse triggering was the most prevalent reverse triggering phenotype. Reverse triggering with a greater EAdi peak and EAdi slope and a longer neural inspiratory time was associated with reverse triggering leading to breath stacking.

### Criteria to define PIDCs

The phrenic nerve activates the diaphragm through three distinct phases of activity: inspiratory, post-inspiratory, and rest (expiration) [28]. During the inspiratory phase, its activity starts with a synchronized onset of discharge that steadily accumulates to maximum but suddenly ends with a complete breakdown [28]. During this phase, an active shortening of its fibers occurs, reducing pleural and intrathoracic pressure, generating inspiratory flow and determining the neural inspiratory time [1, 28]. The post-inspiratory phase occurs at the beginning of expiration and consists of a controlled relaxation of the diaphragm [29]. In the third phase, termed rest or expiration, the diaphragm is at rest, displaying only tonic activity.

We only captured the electrical activity of the crural diaphragm. Nevertheless, we acknowledge that morphologically, histologically and anatomically, there are three major regions of the diaphragm—costal, sternal and crural [30, 31]. Although they likely act synergistically, it is unlikely that they behave as a unit. At least in animal models, there are regional differences in shortening and susceptibility to injury [30, 31]. The crural region is 5–6 times thicker than the costal and also arises from a much less mobile origin—upper three lumbar vertebrae. Whereas the anterior costal is often thinner and likely may be more susceptible to a lengthening force. In animals models, more injury was found in this region [30, 31].

We defined the neural inspiratory time from the start of the EAdi activity to its offset. EAdi offset was defined as when it reduces to 70% of its peak activity. We acknowledge there may be discussions about a universally accepted definition of EAdi offset [32]. However, a drop in EAdi to 70% of its peak activity is a threshold often used [25–27, 32–35] based on studies showing it coincides with the end of the inspiration in people breathing spontaneously [26] and during a spontaneous breathing trial in mechanically ventilated patients [25]. Estrada et al. analyzed the detection of EAdi offset compared to the end of inspiration using flow signals and thresholds ranging from 40 to 100% of the maximum EAdi activity [26]. The optimal threshold values ranged between 66 and 77%. In mechanically ventilated patients undergoing a spontaneous breathing trial with a T-piece, Sinderby et al. described that the onset of expiration coincides with a decrease in peak EAdi by about 30% [25]. Hence, the threshold we applied corresponds to the end of the inspiratory time during spontaneous breathing, aligning with the definition of inspiratory phase of phrenic nerve activity [1, 28]. It is also the threshold utilized by NAVA criteria.

### PIDCs and clinical implications

PIDCs were highly prevalent. Almost all patients included in our study had at least some breaths with PIDCs (Fig. 4A). The exact risk of having PIDCs is still unknown [10]. In animal models, it is suggested that PIDCs occurring during reverse-triggering may be associated with diaphragm dysfunction and morphological damage when associated with large efforts [4, 9], whereas they could help preserve diaphragm function when associated with small efforts [8]. By using the neuromuscular coupling as a measure of diaphragm (dys)function in ICU patients, Coiffard et al., recently suggested that PIDCs may impair diaphragm function during mechanical ventilation [4].

It is suggested that PIDCs are associated with an increased risk of diaphragm injury. This is in part because PIDCs may lead to eccentric diaphragm contractions as PIDCs occur when the thoracic volume is decreasing [9–13]. However, muscle fibers and tendons behave as a *unit* with serial compliance. In locomotor muscles, during a contraction, the stretching of the tendon can counteract the shortening of the muscle fiber, creating the false impression of eccentric contraction [36]. Whether a similar phenomenon occurs between the diaphragm muscle fibers and their tendons during PIDCs remains unknown. If this is the case, not all PIDCs would lead to eccentric diaphragmatic contractions. This might help in explain the contradictory findings about PIDCs being either protective or injurious for the diaphragm. Additionally, whether the magnitude of the effort influences whether a PIDC leads to eccentric contraction also remains unclear.

### PIDCs characteristics and associations with ventilator settings

Compared to breaths without PIDCs, PIDCs were characterized by greater inspiratory drive and longer duration of neural inspiratory time, and associated with low tidal volumes, lower inspiratory flow and shorter ventilator inspiratory time. Ventilator settings and inspiratory drive are modifiable factors that can be targeted for the development of management strategies for PIDCs. Although our data does not allow us to infer causal relationship, one can speculate whether changes in ventilator settings have the potential to reduce or abolish PIDCs in patients triggering the ventilator (e.g., increasing ventilator inspiratory time or tidal volume). Using a decelerating flow pattern may allow to increase insufflation time without reducing peak flow. Nevertheless, our data helps us to understand which patients are at high risk of PIDCs and may deserve further monitoring (e.g., of the magnitude of the inspiratory effort).

Proportional modes like NAVA and proportional assist ventilation with load-adjustable gain factors (PAV +) may represent a solution abolishing PIDCs. These modes adjust the amount and timing of support provided by the ventilator based on the patient's own efforts, making ventilation more dynamic and responsive [37]. For instance, during NAVA the ventilator cycle will occur when the EAdi reduces to 70% of its peak by definition, which would abolish PIDCs. Although we have no data from patients ventilated with PAV + , we demonstrated that no PIDC was detected in 95% of breaths under NAVA and PIDCs, when present, were mostly associated with noise in the EAdi signal.

To further confirm the presence or absence of these PIDCs at the bedside, interpretation of the ventilator waveforms may not suffice, and more advanced monitoring techniques may be necessary (e.g., EAdi or esophageal pressure) [10]. Furthermore, considering that the risk/benefit of PIDCs appears to correlate with the magnitude of effort, measuring the effort associated with these breaths at the bedside could inform decision-making. Nowadays, assessing the effort during a breath is easily achievable at the bedside, such as through end-expiratory occlusion maneuvers (i.e., Pocc) [38]. Consequently, the potential risks or benefits could be addressed directly at the bedside.

### Prevalence of reverse triggering phenotypes

Mid-cycle reverse triggering was the predominant phenotype. Reverse triggering phenotypes were characterized by differences in the neural inspiratory time and the duration of the ventilator inspiratory time. As our study was observational, we cannot draw causal conclusions about whether changing ventilator settings would alter the phenotype or affect the timing, duration or magnitude of the EAdi during reverse triggering breaths. However, this is a subject that deserves further investigation, as different reverse triggering phenotypes are associated with different physiological consequences [3, 7]. For instance, reverse triggering leading to breath stacking, which we classified as a specific phenotype because it has particular consequences (e.g., breath stacking), is the phenotype with the highest potential to cause lung injury and diaphragm dysfunction—it was associate with greater EAdi slope and EAdi peak (potentially large inspiratory efforts). Also, various degrees of PIDCs and the magnitude of the inspiratory effort associated with reverse triggering is, at least in animal models, associated with the development of diaphragm dysfunction [3, 8, 39].

### Limitations and strengths

First, this is a secondary analysis of a prospective observational study. As such, the associations observed in the data do not imply causation. Second, it was a single center study, which may limit the generalizability of the results due to internal protocols for managing mechanical ventilation. Nevertheless, our center follows principles for lung-protective ventilation which are widely used worldwide. Third, data were collected for a maximum of 5 days or until extubation, death, or recovery of significant diaphragm activity, defined in the DIVIP study as a mean EAdi peak of 7µv. This limited duration may not capture the entire course of mechanical ventilation and could potentially miss important changes or trends over time. Fourth, the number of recordings per patient varied including some patients contributing to one while other patients had five recordings. To account for this, we used mixed models considering the patient’s ID as a random effect in our statistical analysis. Fifth, we have no measurement of the magnitude of the effort in our study. Although within patients a greater EAdi is expected to be associated with greater effort, this relationship is non-linear and varies between patients. Therefore, the magnitude of the effort cannot be inferred from our data. Sixth, our study focused on the electrical activity of the diaphragm. During respiratory distress, extra-diaphragmatic respiratory muscles play an important role in supporting (or even substituting) the diaphragm role as main muscle of inspiration [40]. Measurements of extra-diaphragmatic respiratory muscle activity could have provided additional information to our study. Seventh, breaths were used as a unit of measurement, which allowed us to have a large number of data points for statistical comparisons. This is advantageous as it provides substantial power to detect statistically significant between groups differences. However, this can also lead to the detection of small differences as statistically significant, which may not be clinically relevant. To address this, we considered the clinical relevance of the differences, not just their statistical significance, when interpreting our data. Lastly, the cycling criteria of the ventilator in pressure support can interfere in the occurrence of PIDCs. Unfortunately, this information was not available to us.

## Conclusion

We revealed a high prevalence of PIDCs in mechanically ventilated patients. These contractions were particularly common during reverse triggering and present in about 20% of the breaths triggered by the patient under assist-control or pressure support ventilation. PIDCs were associated with specific ventilator settings that can be potentially modifiable as well as patient characteristics. Proportional modes like NAVA represent a solution abolishing PIDCs. Our results highlight the potential for future studies to develop strategies to manage PIDCs and reverse triggering in critically ill patients under mechanical ventilation.

## Data Availability

No datasets were generated or analysed during the current study.
